# Association between convalescent plasma treatment and mortality in COVID-19: a collaborative systematic review and meta-analysis of randomized clinical trials

**DOI:** 10.1186/s12879-021-06829-7

**Published:** 2021-11-20

**Authors:** Cathrine Axfors, Perrine Janiaud, Andreas M. Schmitt, Janneke van’t Hooft, Emily R. Smith, Noah A. Haber, Akin Abayomi, Manal Abduljalil, Abdulkarim Abdulrahman, Yeny Acosta-Ampudia, Manuela Aguilar-Guisado, Farah Al-Beidh, Marissa M. Alejandria, Rachelle N. Alfonso, Mohammad Ali, Manaf AlQahtani, Alaa AlZamrooni, Juan-Manuel Anaya, Mark Angelo C. Ang, Ismael F. Aomar, Luis E. Argumanis, Alexander Averyanov, Vladimir P. Baklaushev, Olga Balionis, Thomas Benfield, Scott Berry, Nadia Birocco, Lynn B. Bonifacio, Asha C. Bowen, Abbie Bown, Carlos Cabello-Gutierrez, Bernardo Camacho, Adrian Camacho-Ortiz, Sally Campbell-Lee, Damon H. Cao, Ana Cardesa, Jose M. Carnate, German Jr. J. Castillo, Rossana Cavallo, Fazle R. Chowdhury, Forhad U. H. Chowdhury, Giovannino Ciccone, Antonella Cingolani, Fresthel Monica M. Climacosa, Veerle Compernolle, Carlo Francisco N. Cortez, Abel Costa Neto, Sergio D’Antico, James Daly, Franca Danielle, Joshua S. Davis, Francesco Giuseppe De Rosa, Justin T. Denholm, Claudia M. Denkinger, Daniel Desmecht, Juan C. Díaz-Coronado, Juan A. Díaz Ponce-Medrano, Anne-Françoise Donneau, Teresita E. Dumagay, Susanna Dunachie, Cecile C. Dungog, Olufemi Erinoso, Ivy Mae S. Escasa, Lise J. Estcourt, Amy Evans, Agnes L. M. Evasan, Christian J. Fareli, Veronica Fernandez-Sanchez, Claudia Galassi, Juan E. Gallo, Patricia J. Garcia, Patricia L. Garcia, Jesus A. Garcia, Mutien Garigliany, Elvira Garza-Gonzalez, Deonne Thaddeus V. Gauiran, Paula A. Gaviria García, Jose-Antonio Giron-Gonzalez, David Gómez-Almaguer, Anthony C. Gordon, André Gothot, Jeser Santiago Grass Guaqueta, Cameron Green, David Grimaldi, Naomi E. Hammond, Heli Harvala, Francisco M. Heralde, Jesica Herrick, Alisa M. Higgins, Thomas E. Hills, Jennifer Hines, Karin Holm, Ashraful Hoque, Eric Hoste, Jose M. Ignacio, Alexander V. Ivanov, Maike Janssen, Jeffrey H. Jennings, Vivekanand Jha, Ruby Anne N. King, Jens Kjeldsen-Kragh, Paul Klenerman, Aditya Kotecha, Fiorella Krapp, Luciana Labanca, Emma Laing, Mona Landin-Olsson, Pierre-François Laterre, Lyn-Li Lim, Jodor Lim, Oskar Ljungquist, Jorge M. Llaca-Díaz, Concepción López-Robles, Salvador López-Cárdenas, Ileana Lopez-Plaza, Josephine Anne C. Lucero, Maria Lundgren, Juan Macías, Sandy C. Maganito, Anna Flor G. Malundo, Rubén D. Manrique, Paola M. Manzini, Miguel Marcos, Ignacio Marquez, Francisco Javier Martínez-Marcos, Ana M. Mata, Colin J. McArthur, Zoe K. McQuilten, Bryan J. McVerry, David K. Menon, Geert Meyfroidt, Ma. Angelina L. Mirasol, Benoît Misset, James S. Molton, Alric V. Mondragon, Diana M. Monsalve, Parastoo Moradi Choghakabodi, Susan C. Morpeth, Paul R. Mouncey, Michel Moutschen, Carsten Müller-Tidow, Erin Murphy, Tome Najdovski, Alistair D. Nichol, Henrik Nielsen, Richard M. Novak, Matthew V. N. O’Sullivan, Julian Olalla, Akin Osibogun, Bodunrin Osikomaiya, Salvador Oyonarte, Juan M. Pardo-Oviedo, Mahesh C. Patel, David L. Paterson, Carlos A. Peña-Perez, Angel A. Perez-Calatayud, Eduardo Pérez-Alba, Anastasia Perkina, Naomi Perry, Mandana Pouladzadeh, Inmaculada Poyato, David J. Price, Anne Kristine H. Quero, Md. M. Rahman, Md. S. Rahman, Mayur Ramesh, Carolina Ramírez-Santana, Magnus Rasmussen, Megan A. Rees, Eduardo Rego, Jason A. Roberts, David J. Roberts, Yhojan Rodríguez, Jesús Rodríguez-Baño, Benjamin A. Rogers, Manuel Rojas, Alberto Romero, Kathryn M. Rowan, Fabio Saccona, Mehdi Safdarian, Maria Clariza M. Santos, Joe Sasadeusz, Gitana Scozzari, Manu Shankar-Hari, Gorav Sharma, Thomas Snelling, Alonso Soto, Pedrito Y. Tagayuna, Amy Tang, Geneva Tatem, Luciana Teofili, Steven Y. C. Tong, Alexis F. Turgeon, Januario D. Veloso, Balasubramanian Venkatesh, Yanet Ventura-Enriquez, Steve A. Webb, Lothar Wiese, Christian Wikén, Erica M. Wood, Gaukhar M. Yusubalieva, Kai Zacharowski, Ryan Zarychanski, Nina Khanna, David Moher, Steven N. Goodman, John P. A. Ioannidis, Lars G. Hemkens

**Affiliations:** 1 Meta-Research Innovation Center at Stanford (METRICS), Stanford University, Stanford, USA; 2 Department for Women’s and Children’s Health, Uppsala University, Uppsala, Sweden; 3 Department of Clinical Research, University Hospital Basel, University of Basel, Spitalstrasse 12, 4031 Basel, Switzerland; 4 Department of Medical Oncology, University of Basel, Basel, Switzerland; 5 Amsterdam University Medical Center, Amsterdam University, Amsterdam, The Netherlands; 6 Department of Global Health, Milken Institute School of Public Health, The George Washington University, Washington, USA; 7 Lagos State Ministry of Health, Lagos, Nigeria; 8 Internal Medicine, Bahrain Defence Force Hospital, Riffa, Bahrain; 9 Medical Team, National Task Force for Combating the Coronavirus (COVID19), Riffa, Bahrain; 10 Mohammed Bin Khalifa Cardiac Centre, Awali, Bahrain; 11 Center for Autoimmune Diseases Research (CREA), Universidad del Rosario, Bogotá, Colombia; 12 Infectious Diseases, Microbiology and Preventive Medicine Unit, Hospital Universitario Virgen del Rocío, Seville, Spain; 13 Surgery and Cancer, Anaesthetics, Pain Medicine and Intensive Care, Imperial College London, London, UK; 14 Department of Medicine, Division of Infectious Diseases, University of the Philippines-Philippine General Hospital, Manila, Philippines; 15 Department of Medicine, Division of Hematology, University of the Philippines-Philippine General Hospital, Manila, Philippines; 16 Centre for Tropical Medicine and Global Health, Nuffield Department of Medicine, University of Oxford, Oxford, UK; 17 Microbiology, Infectious Diseases, Bahrain Defence Force Hospital, Riffa, Bahrain; 18 Microbiology, Royal College of Surgeons in Ireland-Medical University in Bahrain, Riffa, Bahrain; 19 Internal Medicine, Salmaniya Medical Complex, Manama, Bahrain; 20 Department of Laboratories, Division of Blood Bank, University of the Philippines-Philippine General Hospital, Manila, Philippines; 21 Department of Internal Medicine, Hospital Universitario San Cecilio, Granada, Spain; 22 Banco de Sangre, Instituto Nacional de Enfermedades Neoplásicas, Lima, Peru; 23 Pulmonary Division, Federal Scientific and Clinical Center of Specialized Medical Care and Medical Technologies of the Federal Medical and Biological Agency, Moscow, Russian Federation; 24 Fundamental Medicine Department, Pulmonology Scientific and Research Institute under Federal Medical and Biological Agency, Moscow, Russian Federation; 25 Cell Culture Laboratory, Biomedical Research, Federal Scientific and Clinical Center of Specialized Medical Care and Medical Technologies of the Federal Medical and Biological Agency, Moscow, Russian Federation; 26 Laboratory of Personalized Medicine, Pulmonology Scientific and Research Institute under Federal Medical and Biological Agency, Moscow, Russian Federation; 27 Center for Research and Disruption of Infectious Diseases, Department of Infectious Diseases, Copenhagen University Hospital-Amager and Hvidovre, Hvidovre, Denmark; 28 Berry Consultants, Austin, USA; 29 Department of Oncology, University Hospital Città della Salute e della Scienza di Torino, Turin, Italy; 30 Menzies School of Health Research, Casuarina, Australia; 31 Wesfarmers Centre for Vaccines and Infectious Diseases, Telethon Kids Institute, University of Western Australia, Nedlands, Australia; 32 Department of Infectious Diseases, Perth Children’s Hospital, Nedlands, Australia; 33 Rare and Imported Pathogens Laboratory, Public Health England, Porton Down, UK; 34 Department Research in Virology and Mycology, Instituto Nacional de Enfermedades Respiratorias, Mexico City, Mexico; 35 Instituto Distrital de Ciencia Biotecnología e Investigación en Salud (IDCBIS), Bogotá, Colombia; 36 Department of Infectious Diseases, Universidad Autónoma de Nuevo León, Monterrey, Mexico; 37 Pathology, University of Illinois at Chicago, Chicago, USA; 38 Department of Medicine, Division of Nephrology, Henry Ford Hospital, Detroit, USA; 39 Clinical Department, Red Andaluza de Diseño y Traslacion de Terapias Avanzadas, Sevilla, Spain; 40 Department of Laboratories, University of the Philippines-Philippine General Hospital, Manila, Philippines; 41 Department of Laboratory Medicine, Unit of Microbiology and Virology, University Hospital Città della Salute e della Scienza di Torino, Turin, Italy; 42 Internal Medicine, Bangabandhu Sheikh Mujib Medical University, Dhaka, Bangladesh; 43 Internal Medicine, Dhaka Medical College, Dhaka, Bangladesh; 44 Department of Quality and Safety in Health Care, Unit of Clinical Epidemiology, University Hospital Città della Salute e della Scienza di Torino, Turin, Italy; 45 Infectious Disease, Fondazione Policlinico Universitario A. Gemelli IRCCS, Rome, Italy; 46 Department of Medical Microbiology, University of Philippines Manila, Manila, Philippines; 47 Blood Services, Belgian Red Cross-Flanders, Mechelen, Belgium; 48 Faculty of Medicine and Health Sciences, Ghent University, Ghent, Belgium; 49 Instituto D’Or de Pesquisa e Ensino (IDOR), São Paulo, Brazil; 50 Department of Laboratory Medicine, Unit of Transfusion Medicine, University Hospital Città della Salute e della Scienza di Torino, Turin, Italy; 51 Australian Red Cross Lifeblood, Melbourne, Australia; 52 Department of Laboratory Medicine, Blood Bank, University Hospital Città della Salute e della Scienza di Torino, Turin, Italy; 53 Department of Medical Sciences, Unit of Infective Diseases, University Hospital Città della Salute e della Scienza di Torino, Turin, Italy; 54 Victorian Infectious Diseases Service, The Royal Melbourne Hospital, Melbourne, Australia; 55 Doherty Department, University of Melbourne, The Peter Doherty Institute for Infection and Immunity, Melbourne, Australia; 56 Center of Infectious Diseases, Division of Tropical Medicine, Heidelberg University Hospital, Heidelberg, Germany; 57 Animal Pathology, Liège University, Liège, Belgium; 58 Deparment of Internal Medicine, Universidad CES, Medellín, Colombia; 59 Centro Medico Naval, Mexico City, Mexico; 60 Public Health Department, Biostatistic, Liège University, Liège, Belgium; 61 Lagos State University Teaching Hospital, Lagos, Nigeria; 62 Clinical, Research and Development, NHS Blood and Transplant, Oxford, UK; 63 Radcliffe Department of Medicine and BRC Haematology Theme, University of Oxford, Oxford, UK; 64 Clinical Trials Unit, NHS Blood and Transplant, Cambridge, UK; 65 CENETEC (National Center for Health Technology Excellence), Mexico City, Mexico; 66 Blood Bank, Centro Médico Naval and FES Iztacala UNAM, Mexico City, Mexico; 67 Genoma CES, Universidad CES, Medellín, Colombia; 68 Facultad de Salud Pública y Administración, Universidad Peruana Cayetano Heredia, Lima, Peru; 69 Servicio de Hemoterapia y Banco de Sangre, Instituto Nacional de Salud del Niño San Borja, Lima, Peru; 70 Department of Haematology, Centro Transfusional Tejidos y Celulas de Granada, Granada, Spain; 71 Department of Infectious Diseases, Hospital Universitario Puerta del Mar, Cádiz, Spain; 72 Department of Hematology, Universidad Autónoma de Nuevo León, Monterrey, Mexico; 73 Intensive Care, Imperial College Healthcare NHS Trust, London, UK; 74 Immunohematology, Liège University Hospital, Liège, Belgium; 75 ANZIC-RC, School of Public Health and Preventive Medicine, Monash University, Melbourne, Australia; 76 Intensive Care Medicine, Cliniques Universitaires de Bruxelles-Erasme, Université Libre de Bruxelles, Brussels, Belgium; 77 The George Institute for Global Health, Sydney and New Delhi, Sydney, Australia; 78 Microbiology Services, NHS Blood and Transplant, London, UK; 79 Department of Biochemistry and Molecular Biology, University of the Philippines, Manila, Philippines; 80 Medicine, Division of Infectious Diseases, Immunology, and International Medicine, University of Illinois at Chicago, Chicago, USA; 81 Medical Research Institute of New Zealand, Wellington, New Zealand; 82 Auckland City Hospital, Auckland, New Zealand; 83 Division of Pulmonary and Critical Care Medicine, Department of Internal Medicine, Henry Ford Hospital, Detroit, USA; 84 Division of Infection Medicine, Department of Clinical Sciences, Lund University, Lund, Sweden; 85 Infectious Diseases, Skåne University Hospital, Lund, Sweden; 86 Blood Transfusion, Sheikh Hasina National Institute of Burn and Plastic Surgery, Dhaka, Bangladesh; 87 Intensive Care Medicine, Gand University Hospital, Gent, Belgium; 88 Department of Neumology and Pulmonology, Hospital Quiron de Marbella, Málaga, Spain; 89 Center for Precision Genome Editing and Genetic Technologies for Biomedicine, Engelhardt Institute of Molecular Biology of the Russian Academy of Sciences, Moscow, Russian Federation; 90 Department of Hematology, Oncology and Rheumatology, Internal Medicine V, University Hospital Heidelberg, Heidelberg, Germany; 91 The George Institute for Global Health, Sydney and New Delhi, New Delhi, India; 92 School of Public Health, Imperial College, London, UK; 93 Prasanna School of Public Health, Manipal Academy of Higher Education, Manipal, India; 94 Clinical Immunology and Transfusion Medicine, University and Regional Laboratories, Region Skåne, Lund, Sweden; 95 Facultad de Medicina, Instituto de Medicina Tropical Alexander Von Humboldt, Universidad Peruana Cayetano Heredia, Lima, Peru; 96 Department of Clinical Sciences, Lund University, Lund, Sweden; 97 Department of Endocrinology, Skåne University Hospital, Lund, Sweden; 98 Intensive Care Medicine, Saint-Luc University Hospital, Brussels, Belgium; 99 Eastern Health, Box Hill, Australia; 100 Clinical Sciences, Clinical Infection Medicine, Lund University, Malmo, Sweden; 101 Department of Clinical Pathology, Universidad Autónoma de Nuevo León, Monterrey, Mexico; 102 Department of Infectious Diseases, Hospital Universitario Virgen de Las Nieves, Granada, Spain; 103 Department of Infectious Diseases, Hospital Universitario de Jerez de La Frontera, Jerez de la Frontera, Spain; 104 Division of Transfusion Medicine, Department of Pathology, Henry Ford Hospital, Detroit, USA; 105 Department of Infectious Diseases, Hospital Universitario de Valme, Sevilla, Spain; 106 Epidemiology and Biostatistics Research Group, Universidad CES, Medellín, Colombia; 107 Department of Internal Medicine, Hospital Quiron de Malaga, Málaga, Spain; 108 Department of Infectious Diseases, Hospital Regional Universitario de Malaga, Málaga, Spain; 109 Infectious Disease Unit, Hospital Universitario Juan Ramon Jimenez, Huelva, Spain; 110 Department of Internal Medicine, Hospital San Juan de Dios del Aljarafe, Bormujos, Spain; 111 Department of Critical Care Medicine, Auckland City Hospital, Auckland, New Zealand; 112 Department of Epidemiology and Preventive Medicine, Monash University, Melbourne, Australia; 113 Department of Haematology, Monash Health, Melbourne, Australia; 114 Division of Pulmonary, Allergy, and Critical Care Medicine, University of Pittsburgh School of Medicine, Pittsburgh, USA; 115 University Division of Anaesthesia, Addenbrooke’s Hospital Cambridge, University of Cambridge, Cambridge, UK; 116 Intensive Care Medicine, Leuven University Hospital, Leuven, Belgium; 117 Intensive Care Medicine, Liège University Hospital, Liège, Belgium; 118 Western Health, Melbourne, Australia; 119 Department of Medicine, Division of Allergy and Immunology, University of the Philippines-Philippine General Hospital, Manila, Philippines; 120 Thalassemia and Hemoglobinopathy Research Center, Ahvaz Jundishapur University of Medical Sciences, Ahvaz, Iran; 121 Thalassemia and Hemoglobinopathy Research Center, Health Research Institute, Ahvaz, Iran; 122 Middlemore Hospital, Auckland, New Zealand; 123 Clinical Trials Unit, Intensive Care National Audit and Research Centre, London, UK; 124 Blood Services, Red Cross, Suarlée, Belgium; 125 School of Medicine and Medical Sciences, University College Dublin-Clinical Research Centre, University College Dublin, Dublin, Ireland; 126 Australian and New Zealand Intensive Care Research Centre, School of Public Health and Preventive Medicine, Monash University, Melbourne, Australia; 127 Intensive Care Medicine, Alfred Health, Melbourne, Australia; 128 Department of Infectious Diseases, Aalborg University Hospital, Aalborg, Denmark; 129 Institute of Clinical Pathology and Medical Research, NSW Health Pathology, Westmead, Australia; 130 Centre for Infectious Diseases and Microbiology, Westmead Hospital, Westmead, Australia; 131 Faculty of Medicine and Health, University of Sydney, Sydney, Australia; 132 Department of Internal Medicine, Hospital Costa del Sol, Málaga, Spain; 133 College of Medicine, University of Lagos, Lagos, Nigeria; 134 Department of Infectious Diseases, Centro Transfusional Tejidos y Celulas de Sevilla, Sevilla, Spain; 135 Hospital Universitario Mayor Méderi, Universidad del Rosario, Bogotá, Colombia; 136 Centre for Clinical Research, Faculty of Medicine, The University of Queensland, Herston, Australia; 137 Adult Intensive Care Unit, Centro Medico Naval, Mexico City, Mexico; 138 Acute Medicine, Head ICU, Hospital General de Mexico, Mexico City, Mexico; 139 Emergency Medicine Department, School of Medicine, Ahvaz Jundishapur University of Medical Sciences, Ahvaz, Iran; 140 Department of Internal Medicine, Hospital Universitario Torrecardenas, Almería, Spain; 141 Doherty Department, University of Melbourne, Peter Doherty Institute for Infection and Immunity, Melbourne, Australia; 142 Centre for Epidemiology and Biostatistics, Melbourne School of Population and Global Health, University of Melbourne, Melbourne, Australia; 143 Pharmacology, Bangabandhu Sheikh Mujib Medical University, Dhaka, Bangladesh; 144 Department of Internal Medicine, Division of Infectious Diseases, Henry Ford Hospital, Detroit, USA; 145 Department of Medicine, University of Melbourne, Melbourne, Australia; 146 Royal Melbourne Hospital, Melbourne Health, Melbourne, Australia; 147 Departments of Pharmacy and Intensive Care Medicine, Royal Brisbane and Women’s Hospital, Brisbane, Australia; 148 Division of Anaesthesiology Critical Care Emergency and Pain Medicine, Nîmes University Hospital, University of Montpellier, Nîmes, France; 149 Clinical and Research and Development, NHS Blood and Transplant, Oxford, UK; 150 Clinica del Occidente, Bogotá, Colombia; 151 Infectious Diseases and Clinical Microbiology Unit, Hospital Universitario Virgen Macarena, Sevilla, Spain; 152 Department of Medicine, University of Sevilla-IBiS, Sevilla, Spain; 153 Monash University, Melbourne, Australia; 154 Monash Health, Melbourne, Australia; 155 Department of Infectious Diseases, Hospital Universitario de Puerto Real, Cádiz, Spain; 156 Intensive Care National Audit and Research Centre (ICNARC), London, UK; 157 Nanotechnology Research Center, Ahvaz Jundishapur University of Medical Sciences, Ahvaz, Iran; 158 Department of Medical Hospital Direction, Unit of Medical Direction, University Hospital Città della Salute e della Scienza di Torino, Turin, Italy; 159 St Thomas’ Hospital, Guy’s and St Thomas’ NHS Foundation Trust, London, UK; 160 School of Immunology and Microbial Sciences, Kings College London, London, UK; 161 Sydney School of Public Health, University of Sydney, Camperdown, Australia; 162 Sydney Children’s Hospital Network, Westmead, Australia; 163 Facultad de Medicina Humana, Instituto de Investigación en Ciencias Biomédicas (INICIB), Universidad Ricardo Palma, Lima, Peru; 164 Department of Internal Medicine, Hospital Nacional Hipolito Unanue, Lima, Peru; 165 Public Health Sciences, Henry Ford Hospital, Detroit, USA; 166 Transfusion Medicine, Fondazione Policlinico Universitario A. Gemelli IRCCS, Rome, Italy; 167 Victorian Infectious Diseases Service, The Royal Melbourne Hospital, at the Peter Doherty Institute for Infection and Immunity, Melbourne, Australia; 168 Department of Infectious Diseases, The University of Melbourne at the Peter Doherty Institute for Infection and Immunity, Melbourne, Australia; 169 Department of Anesthesiology and Critical Care Medicine, Division of Critical Care Medicine, Université Laval, Quebec City, QC Canada; 170 Faculty of Medicine, University of New South Wales, Sydney, Australia; 171 Wesley and Princess Alexandra Hospitals, University of Queensland, Brisbane, Australia; 172 Blood Bank, Centro Medico Naval, Mexico City, Mexico; 173 St John of God Hospital, Subiaco, Subiaco, Australia; 174 Department of Infectious Diseases, Zealand University Hospital, Roskilde, Denmark; 175 Department of Clinical Haematology, Monash Health, Melbourne, Australia; 176 Department of Anesthesiology, Intensive Care Medicine and Pain Therapy, University Hospital Frankfurt, Goethe University, Frankfurt, Germany; 177 Department of Internal Medicine, Critical Care and Hematology/Medical Oncology, University of Manitoba, Winnipeg, Canada; 178 Division of Infectious Diseases and Hospital Hygiene and Infection Biology Laboratory, University Hospital Basel and University of Basel, Basel, Switzerland; 179 Centre for Journalology, Clinical Epidemiology Program, Ottawa Hospital Research Institute, Ottawa, Canada; 180 Stanford University School of Medicine, Stanford, USA; 181 Department of Epidemiology and Population Health, Stanford University School of Medicine, Stanford, USA; 182 Department of Biomedical Data Science, Stanford University School of Medicine, Stanford, USA; 183 Stanford Prevention Research Center, Department of Medicine, Stanford University, Stanford, USA; 184 Meta-Research Innovation Center Berlin (METRIC-B), Berlin Institute of Health, Berlin, Germany

**Keywords:** Meta-analysis, SARS-CoV-2, COVID-19, Convalescent plasma

## Abstract

**Background:**

Convalescent plasma has been widely used to treat COVID-19 and is under investigation in numerous randomized clinical trials, but results are publicly available only for a small number of trials. The objective of this study was to assess the benefits of convalescent plasma treatment compared to placebo or no treatment and all-cause mortality in patients with COVID-19, using data from all available randomized clinical trials, including unpublished and ongoing trials (Open Science Framework, https://doi.org/10.17605/OSF.IO/GEHFX).

**Methods:**

In this collaborative systematic review and meta-analysis, clinical trial registries (ClinicalTrials.gov, WHO International Clinical Trials Registry Platform), the Cochrane COVID-19 register, the LOVE database, and PubMed were searched until April 8, 2021. Investigators of trials registered by March 1, 2021, without published results were contacted via email. Eligible were ongoing, discontinued and completed randomized clinical trials that compared convalescent plasma with placebo or no treatment in COVID-19 patients, regardless of setting or treatment schedule. Aggregated mortality data were extracted from publications or provided by investigators of unpublished trials and combined using the Hartung–Knapp–Sidik–Jonkman random effects model. We investigated the contribution of unpublished trials to the overall evidence.

**Results:**

A total of 16,477 patients were included in 33 trials (20 unpublished with 3190 patients, 13 published with 13,287 patients). 32 trials enrolled only hospitalized patients (including 3 with only intensive care unit patients). Risk of bias was low for 29/33 trials. Of 8495 patients who received convalescent plasma, 1997 died (23%), and of 7982 control patients, 1952 died (24%). The combined risk ratio for all-cause mortality was 0.97 (95% confidence interval: 0.92; 1.02) with between-study heterogeneity not beyond chance (I^2^ = 0%). The RECOVERY trial had 69.8% and the unpublished evidence 25.3% of the weight in the meta-analysis.

**Conclusions:**

Convalescent plasma treatment of patients with COVID-19 did not reduce all-cause mortality. These results provide strong evidence that convalescent plasma treatment for patients with COVID-19 should not be used outside of randomized trials. Evidence synthesis from collaborations among trial investigators can inform both evidence generation and evidence application in patient care.

**Supplementary Information:**

The online version contains supplementary material available at 10.1186/s12879-021-06829-7.

## Introduction

The transfer of plasma from a patient who recovered and is convalescent from coronavirus disease 2019 (COVID-19) to a person currently suffering from the disease aims to create transient passive immunity to combat the active infection. Convalescent plasma treatment has previously been used to treat, e.g., SARS-CoV-1, MERS, and H1N1 influenza [1–4]. Non-randomized studies indicated a beneficial effect on mortality in COVID-19 [5]. However, as stated by the US Food and Drug Administration (FDA) in March, 2020, “although promising, convalescent plasma has not been shown to be effective in every disease studied” [6]. Thousands of patients with COVID-19 worldwide have received convalescent plasma outside of clinical trials. In the US, this has occurred under single-patient emergency investigational new drug authority, as well as the National Expanded Access Protocol [7, 8] and an Emergency Use Authorization (EUA) by the FDA on August 23, 2020 [9]. No authorization has been issued by the European Medicines Agency; however, the European Commission developed guidance for monitored use [10] together with the European Centre for Disease Prevention and Control and the European Blood Alliance, and announced in January 2021 to allocate grants of €36 million to expand plasma collection programs [11].

When the results of the largest convalescent plasma trial enrolling more than 11,000 participants, the Randomised Evaluation of COVID-19 Therapy (RECOVERY) Trial, were published as press release in January 2021, four randomized trials on convalescent plasma had been published in peer-reviewed journals [12–15] and five had been reported in preprints [16–20]. No trial had reported mortality benefits of a convalescent plasma treatment [21]. Subsequently, several other trials have closed their recruitment according to registry entries.

To summarize all available data on mortality effects of convalescent plasma for COVID-19, we conducted a collaborative systematic review and meta-analysis of all published and unpublished randomized clinical trials that are ongoing, discontinued or completed, investigating the effects of convalescent plasma treatment in patients with COVID-19 compared to placebo or no intervention.

## Methods

The study protocol was posted at the Open Science Framework before data collection [22] and not in a review registry. We report the study under consideration of the PRISMA 2020 statement [23].

### Data sources and searches

We identified all eligible trials from ClinicalTrials.gov and the WHO International Clinical Trials Registry Platform [ICTRP] as of September 28, 2020, through the COVID-evidence database. We also searched PubMed, the Cochrane COVID-19 trial registry and the LOVE database [24] for published results (preprints and peer-reviewed journals) as of April 8, 2021 using search strategies with terms related to convalescent plasma and COVID-19 with a standard randomized clinical trials filter (Additional file 1).

### Collaborative approach

For all unpublished and/or ongoing trials identified in the initial search as of September 28, 2020, and during an update by March 1, 2021, trial investigators were invited to provide their data and collaborate (Additional file 2). Investigators were also asked to provide additional details regarding the randomization and allocation concealment procedures for their trial.

### Study selection

We included all trials that reported randomly allocating patients with confirmed or suspected Severe Acute Respiratory Syndrome Coronavirus 2 (SARS-CoV-2) infection to a treatment with convalescent plasma versus placebo or no additional treatment other than the usual local care. We considered all trials that randomized at least one patient in the experimental arm and one patient in the control arm, regardless of the treatment regime for convalescent plasma or standard of care, as long as there were no differences in the treatments used in the arms beyond the convalescent plasma treatment or placebo. Trials could report all-cause mortality at any time point regardless of whether it was the primary outcome or not. We did not put any restrictions on trial status, language, geographical region, or healthcare setting. One reviewer (CA or PJ) screened each record for inclusion and potential duplicates of trials. Deduplication was conducted in R version 3.6.2 (R Foundation for Statistical Computing).

### Data extraction and risk of bias assessment

We extracted the following trial characteristics based on the trial registry record or the publication (where available): descriptions of experimental and control arms, patient setting, eligibility criteria for recipients and donors, study location, blinding, target sample size, trial status. We contacted investigator teams of all trials without published results (Additional file 2) and requested aggregated, trial-level mortality data and confirmation of the descriptive characteristics that we extracted. Each data point was thus collected by two reviewers (CA/PJ and collaborating trial investigators). If several follow-up points were available, we chose the longest. For each treatment arm in a trial, we collected the number of deceased patients and the number of randomized patients (intention-to-treat data). We also collected information on the number of patients without available mortality data (lost to follow-up). Finally, for potentially eligible trials that were not included, we extracted the current recruitment status as of March 1, 2021 from trial registries and asked investigators for confirmation of the status and current accrual.

Two reviewers (CA and PJ) independently assessed the risk of bias of included RCTs using the Cochrane risk of bias tool 2.0 [25]. Disagreements were resolved through discussion. The assessment was done using information reported in the preprints and journal publications or provided by investigators for unpublished trials. Small-study effects were assessed using a funnel plot and Egger’s test. The presence of small-study effects may be suggestive, but not definitive, of publication bias [26].

### Data synthesis and analysis

We prespecified all-cause mortality as our sole outcome. We report absolute numbers, proportions, and treatment effect estimates (risk ratio, RR) with 95% confidence interval (CI). A meta-analysis was performed to combine RRs across all trials using the Hartung-Knapp-Sidik-Jonkman (HKSJ) random-effects model [27] with Paule and Mandel (PM) tau-squared estimator, correcting for zero events in one study arm by adding the reciprocal of the size of the contrasting arm [28]. We expected a large variation in sample size and in the number of outcome events across trials, with a proportion of trials presenting with zero events in one or both arms and therefore the HKSJ-PM method would perform well in terms of equality of weights between trials. Statistical heterogeneity is described with the I^2^-statistic [29]. In 3 multi-arm studies, we considered each eligible comparison separately in the main analysis as prespecified; we also added a sensitivity analysis combining them. A RR < 1 means treatment with convalescent plasma reduced overall mortality.

We conducted sensitivity analyses to assess robustness across meta-analytic approaches using the DerSimonian–Laird and Sidik–Jonkman tau-squared estimators, Mantel–Haenszel random-effects method, Peto’s odds ratio method and profile likelihood method. We also repeated all meta-analytic approaches using the arcsine difference, a variant to the handling of zero events. DerSimonian–Laird is a standard random-effects meta-analysis approach but underestimates uncertainty. The Sidik–Jonkman tau-squared estimator, on the other hand, may yield inflated estimates if heterogeneity is low [30]. The Mantel–Haenszel method performs reasonably well with small and zero event counts, similar to Peto’s odds ratio method or with the arcsine transformation for zero events. The Peto’s odds ratio method is, however, suboptimal in the presence of substantial imbalances in the allocation of patients randomized in the compared arms.

In exploratory subgroup analyses, we stratified trials by (1) publication status (results published in peer-reviewed publications and preprints versus unpublished); (2) patient setting (ICU patients; inpatients with oxygen supplementation; inpatients with or without oxygen supplementation); and (3) antibody titer level (confirmed high-titer versus low-titer or unconfirmed titer). We defined high-titer as S-protein receptor binding domain (RBD)-specific IgG antibody titer of 1:640 or higher, or serum neutralization titer of 1:40 or higher [14]. For studies using the Ortho VITROS SARS-CoV-2 IgG test, which reports a signal-to-cutoff (S/C) value, we defined high titer as S/C > 12 (corresponding to the initial US emergency use authorization) as prespecified. We complemented the high-titer definition with additional information made available in the March 2021 version emergency use authorization [31] (e.g., EUROIMMUN (ratio ≥ 3.5) and Abbott ARCHITECT (S/C ≥ 4.5). We furthermore stratified trials by (4) control type (placebo versus no treatment); (5) timing of treatment (maximum 14 days after symptom onset versus not maximum 14 days after symptom onset); (6) donor pregnancy history (only using donated plasma from men, nulliparous women, or women testing negative for human leukocyte antigen (HLA) antibodies, versus including non-nulliparous women without HLA antibody testing); and (7) donor severity of COVID-19 (moderate or severe disease [e.g., whose infection required hospitalization] versus mild disease) [1, 32]. We added a non-prespecified subgroup analysis stratified by region, pooling high-income countries (Australia, Bahrain, Belgium, Chile, Germany, Italy, Netherlands, New Zealand, Spain, Sweden, United Kingdom, USA) versus middle-income countries (Argentina, Bangladesh, Brazil, China, Colombia, India, Iran, Mexico, Nigeria, Peru, Philippines, Russia) [33]. We also added a non-prespecified subgroup analysis separating trials with early administration of high-titer plasma in hospitalized patients from other trials, given the updated emergency use authorization by the FDA in February 2021 [34] The non-prespecified analyses are further described in Additional file 3; there were no substantial deviations from the protocol. For the subgroup analysis on donor pregnancy status (“Excluding potentially HLA antibody positive persons”) we used the non-prespecified Hartung–Knapp “ad hoc” variance correction [35] (this group included only two very small studies with large imprecision which can provide abnormally anticonservative estimates [36]).

We describe the accumulation of publicly available evidence in a cumulative non-prespecified meta-analysis using the HKSJ random-effects model with PM tau-squared, with published trials ordered by their date of publication or preprint posting, and the unpublished trials added to the model last as one summarized treatment estimate.

We used R version 3.6.2 (the ‘meta’ and ‘metaplus’ packages) for the analyses (R Foundation for Statistical Computing).

### Patient involvement statement

No patients were involved in setting the research question or the outcome measures, nor were they involved in developing plans for design or implementation of the study. No patients were asked to advise on interpretation or writing up of the results. All-cause mortality is selected as an important outcome in COVID-19 research by Core Outcome Set developers that involved patients as a key stakeholder group [37].

## Results

**Fig. 1:**
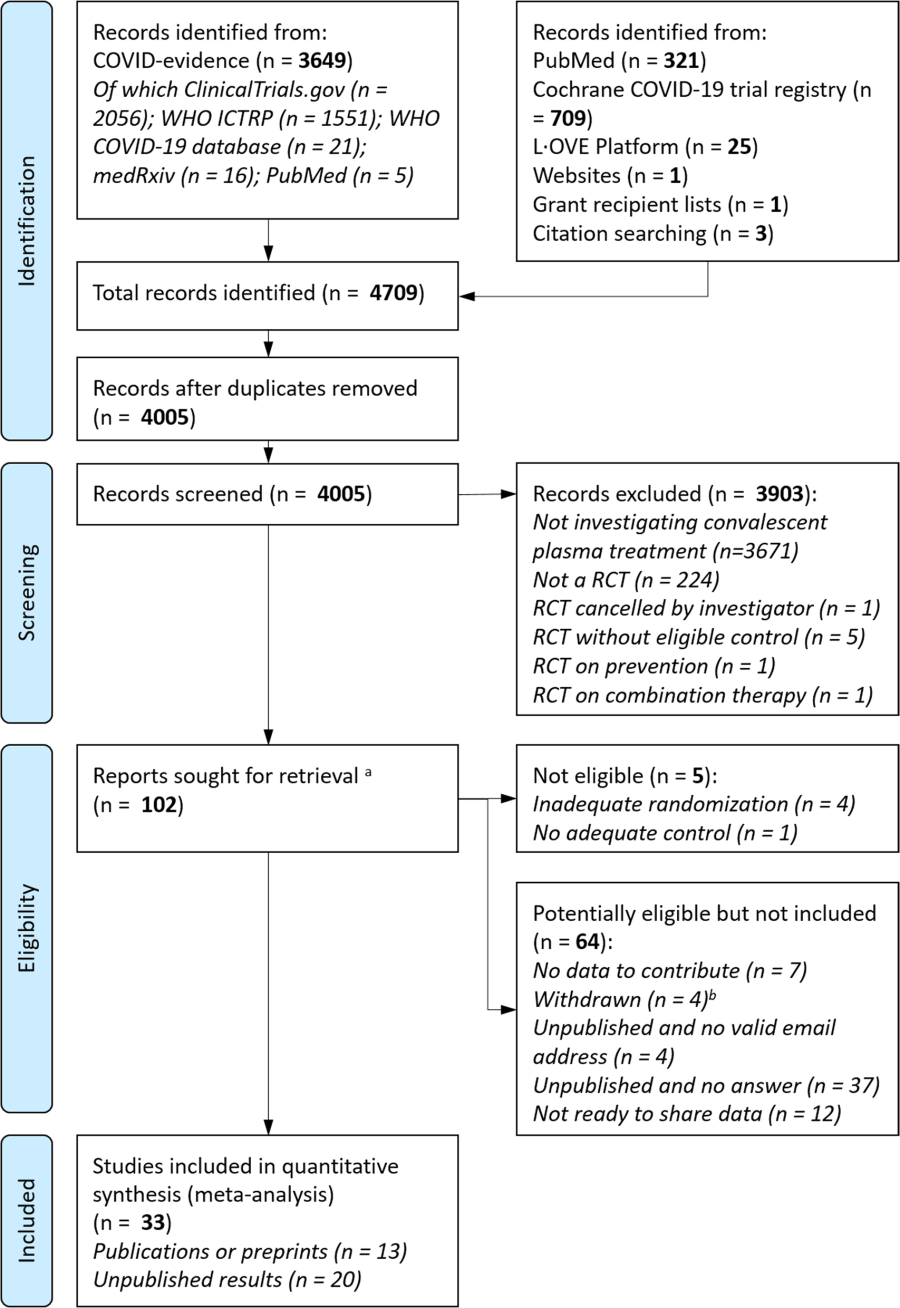
Flowchart of the data collection process. ^a^Of 102 potentially eligible trials, 7 had publications available, and investigators of trials unpublished at the time of our initial search by September 28, 2020 or at an update on March 1, 2021, with a valid email address were contacted (n = 90); of these, 51 responded. All trials that were potentially eligible but not included are described in Additional file 4. ^b^Trials excluded as “withdrawn” are trials labelled as such on the registries (such trials will never be conducted)

Of 4005 unique records identified in trial registries, literature databases, and other repositories, 102 trials were potentially eligible based on the information available (Fig. 1 and Additional file 4). We identified and included 7 already published trials (4 preprints [17–19, 38] and 3 publications) [13–15] at the time of our initial search (September 28, 2020) or at an update (March 1, 2021). In addition, investigators of 90 unpublished trials with a valid email address were contacted, 51 teams responded, 5 trials were confirmed ineligible, and investigators of 26 eligible trials shared their data. Of these, 20 trials are still unpublished, and 5 have been posted as preprints [16, 20, 39] or published in peer-reviewed journals [12, 40] as of April 8, 2021. Since then, the RECOVERY Trial has been published in a peer-review journal [41] and the IRCT20200310046736N trial has been published [42]; resulting in 13 published trials (6 preprints and 7 publications) and 20 unpublished trials included.

We included 33 trials with 16,477 participants (median 66, interquartile range IQR 31 to 120, range 5 to 11,558) (Table 1). Fourteen of these 33 trials were ongoing (42%). Without taking into account the adaptive trials whose final sample size is not fixed (ASCOT, REMAP-CAP and the RECOVERY Trial), 5552 patients were planned to be enrolled in the remaining 30 trials of which 52% (2872/5552) have been included in the meta-analysis.

**Table 1:** Characteristics of included randomized clinical trials of convalescent plasma treatment in COVID-19

Trial acronym and/or registration number	Location	Status	Blinding	Control type	N included (N planned)	Mortality %	Planned mortality time point^a^	Patient setting	Plasma titer, assay (timing of intervention)	Donor severity (HLA antibody status)
*Unpublished*										
LIFESAVER (NCT04374526)	Italy	Recruiting	None	Standard of care	12 (182)	16.67%	28 days	Inpatients with supplemental oxygen	Low titer: ≥ 1:200 S-RBD IgG, EUROIMMUN (Maximum was = < 14 days after symptom onset)	Mild to moderate (Excluding potentially HLA antibody positive persons)
RECOVER (EUCTR2020-001632-10-DE)	Germany	Recruiting	None	Standard of care	90 (174)	13.33%	28, 56 and 84 days	Inpatients with supplemental oxygen	Confirmed high-titer: ≥ 1:80, neutralizing antibody assay (No exclusion based on timing of symptom onset)	Mild to severe (Excluding potentially HLA antibody positive persons)
LACCPT (PACTR202006760881890)	Nigeria	Recruiting	Participant, Care Provider	Placebo (normal saline)	22 (100)	59.09%	3, 5, 7, 9, and 11 days	Inpatients with supplemental oxygen	No minimum cut-off (Maximum was = < 14 days after symptom onset)	Mild to severe (Excluding potentially HLA antibody positive persons)
CPC-SARS (NCT04405310)	Mexico	Recruiting	Participant, Care Provider	Placebo (hartmann plus albumine)	42 (80)	28.57%	15 days	Inpatients with supplemental oxygen	Confirmed high-titer: > 1000 S-RBD IgG, > 1:32 neutralizing, separate in-house assays (Maximum was > 14 days after symptom onset)	Mild to moderate (Excluding potentially HLA antibody positive persons)
NCT04442191	United States	Recruiting	Participant, Care Provider	Placebo (fresh frozen plasma)	14 (50)	7.14%	28 days	Inpatients with supplemental oxygen	Low-titer: S-RBD IgG, Abbott Architect IgG (No exclusion based on timing of symptom onset)	NA (Not excluding potentially HLA antibody positive persons)
NCT04385199	United States	Recruiting	None	Standard of care	30 (30)	16.67%	NR	Inpatients with supplemental oxygen	No minimum cut-off (No exclusion based on timing of symptom onset)	NA (NA)
NCT04403477 (NCT04403477)^b^	Bangladesh	Recruiting	None	Standard of care	30 (60)	18.33%	28 days	Inpatients with supplemental oxygen	Low titer: ≥ 1:160 S-RBD IgG, EUROIMMUN (Maximum was = < 14 days after symptom onset)	Moderate to severe (Excluding potentially HLA antibody positive persons)
PLACO-COVID (NCT04428021)	Italy	Active, not recruiting	Participant, Care Provider, Outcomes Assessor	Standard of care	120 (180)^c^	15.83%	30 days	Inpatients with supplemental oxygen	No minimum cut-off (No exclusion based on timing of symptom onset)	Mild to severe (Excluding potentially HLA antibody positive persons)
REMAP-CAP (NCT02735707)	International	Terminated	None	Standard of care	2014 (N/A)	34.26%	90 days	ICU	Confirmed high-titer: ≥ 1:100 neutralizing or equivalent. In the UK: EUROIMMUN S/C ≥ 6 (No exclusion based on timing of symptom onset)	NA (Excluding potentially HLA antibody positive persons)
ASCOT (NCT04483960)	Australia and New Zealand	Terminated	None	Standard of care	33 (N/A)	9.09%	28 days	Inpatients with or without supplemental oxygen	Confirmed high-titer: ≥ 1:80, neutralizing assay by Walker et al. (2021) (Maximum was = < 14 days after symptom onset)	NA (Excluding potentially HLA antibody positive persons)
Co-CLARITY (NCT04567173)	Philippines	Recruiting	None	Standard of care	25 (136)	0.00%	28 days	Inpatients with or without supplemental oxygen	Low titer: Ortho VITROS S/C ≥ 5 (Maximum was = < 14 days after symptom onset)	Moderate to severe (Excluding potentially HLA antibody positive persons)
NCT04528368	Brazil	Recruiting	None	Standard of care	16 (60)	0.00%	7, 10, 14, 21 and 28 days	Inpatients with or without supplemental oxygen	Low titer: ≥ 1:320 S-RBD IgG, in-house assay (Maximum was = < 14 days after symptom onset)	NA (Excluding potentially HLA antibody positive persons)
CAPSID (NCT04433910)	Germany	Recruiting	None	Standard of care	5 (106)	0.00%	21, 35 and 60 days	ICU	No minimum cut-off (No exclusion based on timing of symptom onset)	NA (Not excluding potentially HLA antibody positive persons)
PERUCONPLASMA (NCT04497324)	Peru	Recruiting	None	Standard of care	25 (100)	16.00%	30 days	Inpatients with supplemental oxygen	Confirmed high-titer: EUROIMMUN S/C > 5.0 (No exclusion based on timing of symptom onset)	Moderate to severe (Excluding potentially HLA antibody positive persons)
NCT04332835	Colombia	Completed	Outcome Assessor	Standard of care	100 (92)	8.00%	7, 14 and 28 days	Inpatients with supplemental oxygen	Confirmed high-titer: IgG ≥ 1/3200 and IgA ≥ 1/800 by EUROIMMUN. All transfused plasma presented neutralizing antibodies ≥ 1/256 (No exclusion based on timing of symptom onset)	Moderate to severe (Excluding potentially HLA antibody positive persons)
CONFIDENT (NCT04558476)	Belgium	Recruiting	None	Standard of care	301 (500)	31.89%	28 and 90 days	ICU	Confirmed high-titer: ≥ 1:320 neutralizing, in-house assay (No exclusion based on timing of symptom onset)	NA (Excluding potentially HLA antibody positive persons)
PC/COVID-19 (NCT04366245)	Spain	Completed	None	Standard of care	41 (72)	2.44%	14 and 28 days	Inpatients with supplemental oxygen	Low titer: Vircell SL (Spain) test, correlates to ≥ 1:320 S-RBD IgG (Maximum was = < 14 days after symptom onset)	Mild to moderate (Excluding potentially HLA antibody positive persons)
COP-COVID-19 (NCT04358783)	Mexico	Recruiting	Participant, Care Provider	Standard of care	31 (30)	35.48%	14 days	Inpatients with supplemental oxygen	Low titer: S/C ≥ 3, Abbott's chemiluminescent microparticle immunoassay for the qualitative detection of IgG (Maximum was = < 14 days after symptom onset)	NA (Excluding potentially HLA antibody positive persons)
NCT04600440	Sweden	Terminated	None	Standard of care	31 (100)	16.13%	90 days	Inpatients with supplemental oxygen	Confirmed high-titer: ≥ 1:40 neutralizing, in-house assay (No exclusion based on timing of symptom onset)	Mild to moderate (Excluding potentially HLA antibody positive persons)
CCAP-2 (NCT04345289)	Denmark	Terminated	Participant, Care Provider, Outcome Assessor	Placebo (normal saline)	144 (1,100)	11.11%	7, 14, 21, 28 and 90 days	Inpatients with or without supplemental oxygen	Other titer level^d^ (No exclusion based on timing of symptom onset)	NA (Excluding potentially HLA antibody positive persons)
*Published*										
ChiCTR2000029757	China	Terminated	None	Standard of care	103 (200)	19.42%	28 days	Inpatients with supplemental oxygen	Confirmed high-titer: ≥ 1:640 S-RBD IgG, in-house assay (NA)	NA (NA)
NCT04342182	Netherlands	Terminated	None	Standard of care	86 (426)	19.77%	60 days	Inpatients with or without supplemental oxygen	Low titer: ≥ 1:400 S-RBD IgG, assay missing (No exclusion based on timing of symptom onset)	NA (Excluding potentially HLA antibody positive persons)
NCT04392414	Russia	Completed	None	Placebo (fresh frozen plasma)	66 (60)	6.06%	30 days	Inpatients with or without supplemental oxygen	Confirmed high-titer: ≥ 1:1000 S-RBD IgG, in-house assay (Maximum was = < 14 days after symptom onset)	Moderate to severe (Not excluding potentially HLA antibody positive persons)
ConPlas-19 (NCT04345523)	Spain	Terminated	None	Standard of care	81 (278)	4.94%	29 days	Inpatients with or without supplemental oxygen	Confirmed high-titer: > 1:80, neutralizing assay (Maximum was = < 14 days after symptom onset)	NA (NA)
PLACID (CTRI/2020/04/024775)	India	Completed	None	Standard of care	464 (452)	14.01%	28 days	Inpatients with supplemental oxygen	No minimum cut-off (No exclusion based on timing of symptom onset)	Mild to moderate (Excluding potentially HLA antibody positive persons)
ILBS-COVID-02 (NCT04346446)	India	Completed	None	Placebo (fresh frozen plasma)	31 (40)	3.23%	28 days	Inpatients with supplemental oxygen	No minimum cut-off (No exclusion based on timing of symptom onset)	NA (Excluding potentially HLA antibody positive persons)
NCT04356534	Bahrain	Completed	None	Standard of care	40 (40)	7.50%	28 days	Inpatients with supplemental oxygen	No minimum cut-off (Maximum was = < 14 days after symptom onset)	Moderate to severe (Excluding potentially HLA antibody positive persons)
PLASM-AR (NCT04383535)	Argentina	Completed	Participant, Care Provider	Placebo (normal saline)	333 (333)	11.11%	30 days	Inpatients with or without supplemental oxygen	Confirmed high-titer: ≥ 1:800 S-RBD IgG, COVIDAR IgG test (No exclusion based on timing of symptom onset)	Mild to moderate (Excluding potentially HLA antibody positive persons)
PICP19 (CTRI/2020/05/025209)	India	Completed	None	Standard of care	80 (80)	30.00%	30 days	Inpatients with supplemental oxygen	No minimum cut-off (Maximum was = < 14 days after symptom onset)	NA (Excluding potentially HLA antibody positive persons)
NCT04479163	Argentina	Terminated	Participant, Care Provider	Placebo (normal saline)	160 (210)	3.75%	25 days	Outpatients	Confirmed high-titer: > 1:1000 (COVIDAR IgG, Instituto Leloir, Argentina) (Maximum was = < 14 days after symptom onset)	NA (NA)
RECOVERY Trial (NCT04381936)	United Kingdom	Terminated	None	Standard of care	11,558 (N/A)	24.28%	28 days	Inpatients with or without supplemental oxygen	Confirmed high-titer: ≥ 1:100 neutralizing (EUROIMMUN IgG ELISA S/C ≥ 6.0) (No exclusion based on timing of symptom onset)	NA (Excluding potentially HLA antibody positive persons)
NCT04359810	United States	Completed	Participant, Outcomes Assessor	Placebo (fresh frozen plasma)	223 (219)	16.59%	28 days	Inpatients with or without supplemental oxygen	Low titer: ≥ 1:400 S-RBD IgG, in-house assay (No exclusion based on timing of symptom onset)	NA (NA)
IRCT20200310046736N1^e^	Iran	Completed	Participant, Outcome Assessor	Standard of care	62 (62)	12.90%	60 days	Inpatients with supplemental oxygen	No minimum cut-off (Maximum was = < 14 days after symptom onset)	NA (Excluding potentially HLA antibody positive persons)

*ELISA* enzyme-linked immunosorbent assay, *IgG* immunoglobulin G, *S-RBD* *IgG* spike-protein receptor binding domain-specific IgG antibody titer, *NA* not reported, *S/C* sample to cutoff ratio

^a^For published trials, we report the planned time point (after randomization) stated in the publications and used for our meta-analysis. For unpublished trials, we report the planned time points as reported in the registries and for the meta-analysis, we used the mortality data available at the time-point of the request, which may not reflect the planned time points (or future publications reporting on it)

^b^NCT04403477 compares two different volumes of plasma (a) 400 mL and (b) 200 mL versus standard of care

^c^The multi-arm PLACO-COVID trial includes another experimental arm (N = 60) treated with standard therapy + standard plasma, a treatment not considered in this review

^d^CCAP-2 initially used a EUROIMMUN cutoff of > 3.0, which was changed to > 3.5 after the March 2021 update of the US FDA emergency use authorization with its new guidance. For this meta-analysis, it is categorized as non-high titer

^e^IRCT20200310046736N was published after the last literature search on April 8th, 2021. The trial team made us aware of their publication

The 33 trials were conducted in Europe (n = 12), Asia (n = 8), South America (n = 7), North America (n = 3), Africa (n = 1), Oceania (n = 1) and transcontinental (n = 1). SARS-CoV-2 infection of all enrolled participants was confirmed in all trials except the RECOVERY Trial that also included patients with probable infection. There were 3 trials (9%) with only ICU patients, and 19 trials (58%) where not all patients required intensive care, but all required oxygen. In 10 trials (30%), patients were recruited regardless of intensive care or oxygen requirement and one trial (3%) recruited only outpatients.

All participants received the usual local care. In 14 trials (42%), all patients received convalescent plasma within 14 days since symptom onset. The plasma was confirmed to have high antibody titers in 15 (45%) trials; and was obtained from donors with moderate or severe COVID-19 in 6 trials (18%). Twenty-three trials (70%) excluded women donors who were pregnant or had previously been pregnant (or who did not test negative for HLA antibodies). Patients randomized to the control group received in 24 (74%) trials no additional treatment than the usual local care and in 9 (26%) trials a placebo infusion.

The risk of bias was considered as low for 29 out of the 33 included trials. For 3 trials it was unclear due to inadequate description of the allocation concealment procedure or concerns about open label trials reported without patient flowcharts. Risk of bias was considered high for 1 trial due to missing information about potential protocol deviations (Additional file 5). Loss to follow-up was minimal (0% in 20 trials, ranging from 0.003 to 9% in 13 trials). Assessment of small study effects resulted in a statistically significant Egger’s test (p-value 0.046; Additional file 6).

### Recruitment status of nonincluded trials

We surveyed 64 unpublished potentially eligible trials (i.e. eligibility based on the information provided in the registries) that were not included in this analysis for their current recruitment status. Out of the 64 trials, 14 were not yet recruiting (22%), 33 recruiting (52%), 2 terminated early (3%), 10 completed (16%), and 4 were withdrawn (6%) and one was not identifiable at the trial registry. However, the status of the 47 trials marked as recruiting or not yet recruiting remains unclear since their latest registry update occurred at a median of August 2020 (IQR: May 2020 to December 2020). Investigators of 14 out of the 64 trials (22%) provided current accrual as of February/March, 2021, with a total of 3076 participants recruited out of a total target sample size of 3989 participants (median recruitment 80 participants, IQR 0 to 483; median proportion of target sample 55%, IQR 6 to 100%).

The total target sample size of all unavailable completed or terminated trials was 1457 participants (median 88 participants, IQR 55 to 142). Of the 97 eligible trials (33 included and 64 not included), there is evidence available from at least 20,499 participants, of which at least 4022 participants (20%) are enrolled in unpublished trials that we have not included in this analysis.

### All-cause mortality

**Fig. 2:**
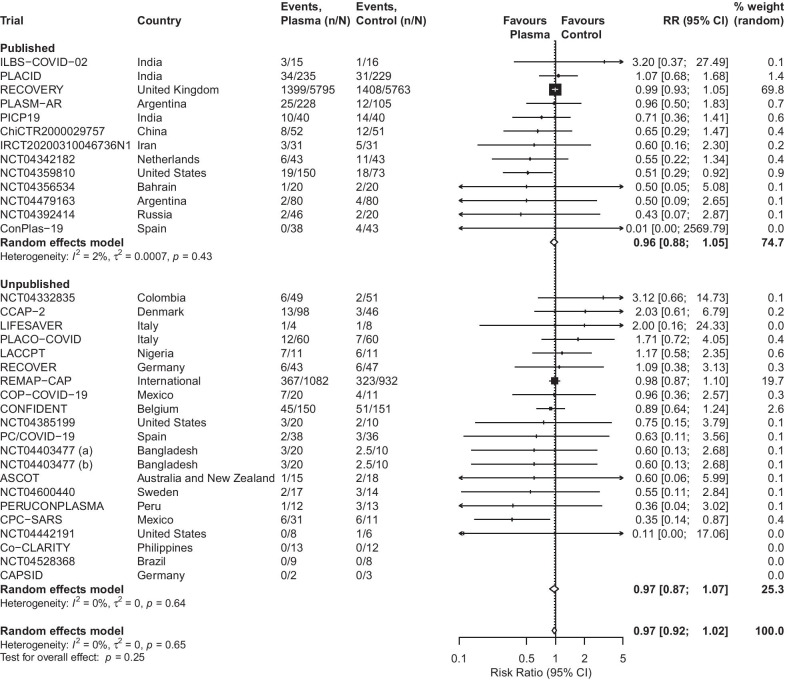
Random effects meta-analysis on the association between convalescent plasma treatment compared to placebo or no treatment and all-cause mortality in patients with COVID-19, stratified by publication status. *CI* confidence interval, *RR* risk ratio. NCT04403477 compares two different volumes of plasma **a** 400 mL and **b** 200 mL versus standard of care. To avoid double counting the control arm, the number of patients and number of events were split equally between the two comparisons

Overall, 3949 of 16,477 patients died (24%). The mortality in patients treated with plasma was 23% (1997/8495) versus 24% (1952/7982) in patients in the various control groups. The mortality rates in the control groups varied considerably ranging from 0 to 54% (median 15% IQR 10 to 25%), with nine trials with a mortality rate of 25% and above in their control groups. The combined RR for all-cause mortality was 0.97 (95% CI [0.92; 1.02]; p-value = 0.25) (Fig. 2). There was no between-study heterogeneity beyond that expected by chance (I^2^ = 0%; tau^2^ = 0, 95% CI [0; 0.12]). In 3 trials including 47 patients, there were zero deaths in both arms. The RECOVERY Trial and the unpublished REMAP-CAP trial accounted for 69.8% and 19.7% of the weight in the meta-analysis, and 70% (11,558/16,477) and 12% (2014/16,477) of the patients included, respectively. The unpublished evidence overall accounted for 25.3% of the weight in the meta-analyses and 3190 of the 16,477 patients included (19%).

**Fig. 3:**
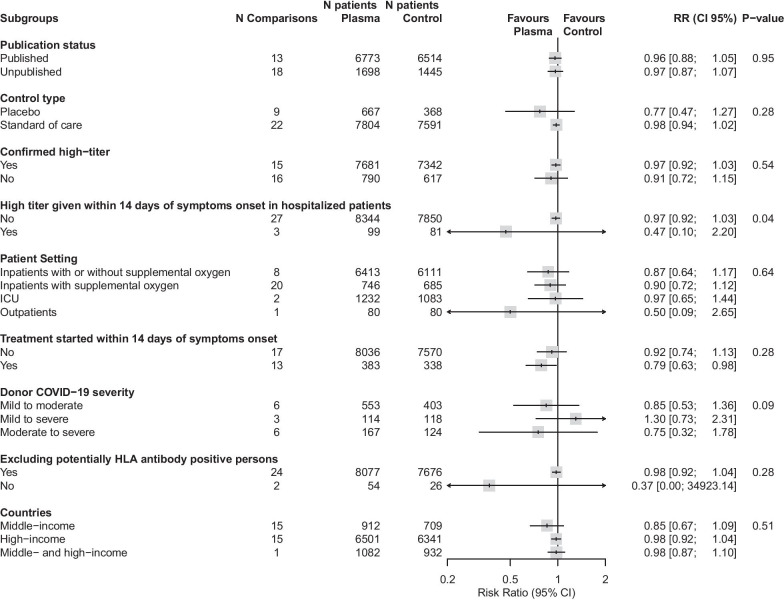
Subgroup analyses. *CI* confidence interval, *ICU* intensive care unit, *RR* risk ratio. Subgroup analyses include only trials with at least one event in one arm (i.e. trials with zero events in both arms were excluded). Number of comparisons differ from number of trials as two trials had more than one comparison. We defined high-titer as S-protein receptor binding domain (RBD)-specific IgG antibody titer of 1:640 or higher, or serum neutralization titer of 1:40 or higher. For studies using the Ortho VITROS SARS-CoV-2 IgG test, which reports a signal-to-cutoff (S/C) value, we defined high titer as S/C > 12 (corresponding to the initial United States Food and Drug Administration emergency use authorization) as prespecified. We complemented the high-titer definition with additional information made available in the March 2021 version emergency use authorization, e.g., EUROIMMUN (ratio ≥ 3.5) and Abbott ARCHITECT (S/C ≥ 4.5)

The sensitivity analyses employing different meta-analytical methods results were compatible with the main analysis (Additional file 7). No potential effect modifiers were detected (Fig. 3).

### Accumulation of evidence in published and unpublished trials

**Fig. 4:**
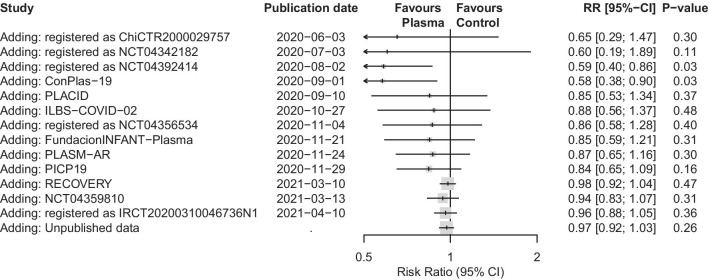
Accumulation of evidence over time (Cumulative meta-analysis). *CI* confidence interval, *RR* risk ratio. Published trials are ordered by their date of publication. For the PLACID trial we used the date when it was first posted as a preprint (September 10th, 2020) before being published in a peer reviewed journal (October 22nd, 2020). Similarly, NCT04479163 was first posted as a preprint (November 21st, 2020) before being published a peer reviewed journal (February 18th, 2021) and RECOVERY Trial was first posted as a preprint (March 10th, 2021) before being published in a peer reviewed journal (May 14th, 2021)

The accumulation of evidence generated through publications and the addition of unpublished data through the collaborative effort was characterized by two major shifts in the treatment effect estimates over time (Fig. 4). For a short period of time, when 4 trials were available, the cumulative meta-analysis suggested a nominally significant benefit (p = 0.03; with limited evidence, however, as transient nominally significant results upon sequential addition of trials can be misleading) [43]. The first shift occurred with the publication of the PLACID trial (before September 10th, 2020 RR 0.58, 95% CI [0.38; 0.90]; with the PLACID trial RR 0.84 95% CI [0.53; 1.34]), and the second shift occurred when the RECOVERY Trial was posted as a preprint (before March 10th, 2021 RR 0.84, 95% CI [0.65; 1.09]; with the RECOVERY trial RR 0.98, 95% CI [0.92; 1.04]). The addition of the unpublished trial evidence greatly increased the precision of the effect estimate (before unpublished trials RR 0.96, 95% CI [0.88; 1.05]; with the unpublished trials RR 0.97, 95% CI [0.92; 1.03]) and also corroborates the findings of the RECOVERY Trial, showing highly similar effects (RECOVERY RR 0.99, 95% CI [0.93; 1.05] versus unpublished combined RR 0.97, 95% CI [0.87; 1.07]).

## Discussion

This meta-analysis of 33 clinical trials, including 16,477 patients with COVID-19, showed that treatment with convalescent plasma did not decrease all-cause mortality and confidence intervals excluded a meaningful clinical effect. This analysis is the largest available body of randomized clinical trial evidence on treatment benefits of convalescent plasma in COVID-19 to-date. There was no indication that the treatment was associated with more or less benefit in patients with different disease severity or with the type of plasma, but data for subgroup analyses were sparse. Only few trials assessed early administration of plasma and further analyses are required to investigate a potential effect modification of the timing of the intervention, and whether patients have already developed their own antibodies by the time of the treatment. The vast majority of trials included patients with moderate or severe COVID-19 who needed hospitalization and it is unclear if the results are applicable to outpatients.

In addition to providing the most complete body of evidence including all available mortality data, our collaborative approach was also driven by the opportunity to allow all trials to publicly share their data regardless of their planned sample size or final results. Beyond reducing research waste, such collaboration of trial investigators and evidence synthesis aims to inform the generation of clinical trial evidence and the application of evidence for clinical care in a timely fashion [44].

The evidence base was dominated by the RECOVERY Trial and REMAP-CAP, which accounted for 89.5% of the weight in the meta-analysis and 82% of the patients included. For both trials, the lack of benefit on mortality outcomes were initially communicated through press releases. Those highly anticipated announcements might have had an impact on the future of clinical trials assessing convalescent plasma. Since February 4, 2021, the emergency use authorization in the US no longer authorizes use in outpatients, patients beyond an early disease stage or of low-titer plasma [34] followed by similar changes in the European Commission’s guidance for monitored use [10]. Although this authorization does not apply to trials, recruitment for trials including such patients or low-titer treatments could become more difficult. Out of the 33 included RCTs, 9 have been terminated early; moreover, out of 64 eligible trials not included, at least four were withdrawn, two terminated early, and more might follow. However, the remaining amount of evidence that is not covered by this analysis is small.

Traditional systematic reviews have many strengths, but they take time and may struggle to capture unpublished data. Others have highlighted the need for an accelerated evidence synthesis regarding the benefits and harms of COVID-19 interventions such as convalescent plasma [45], suggesting a rapid review approach or continuously updated (living) systematic reviews (LSR), particularly ones that incorporate emerging technologies to automate certain aspects of the review process. LSR are valuable [46, 47], but are dependent on traditional availability of data, which can be slower than needed in urgent contexts. Our approach, built on a similar strategy used to investigate hydroxychloroquine/chloroquine [48] was designed to accelerate the evidence synthesis for rapid provision of urgently needed information to guide clinical decision making. We offered investigators the opportunity to share trial results regardless of trial or publication status, which was done only after careful consideration and approval of principal investigators and data steering committees.

Our collaboration focused on aggregated data of one critical outcome, robust to various types of bias: all-cause mortality. We encouraged teams to continue their plans for individual publications, which will display the granularity not captured by our rapid approach, as well as to participate in other collaborations. We are aware of one other international real-time collaboration, the Continuous Monitoring of Pooled International Trials of Convalescent Plasma for COVID-19 Hospitalized Patients (COMPILE) project [49], a highly granular, individual patient data meta-analysis including eight trials [50].

We identified almost a hundred eligible trials that evaluate evidence on convalescent plasma treatment in patients with COVID-19. Among 21 other systematic reviews and meta-analyses on this topic that were available on PubMed as of mid-April, 2021 [5, 46, 47, 51–68], 15 include only 0 to 2 randomized trials alongside observational studies (e.g., two LSR) [46, 47, 51–63] and the two most comprehensive reviews included 10 RCTs [5, 64]. One of the latter meta-analyses [5] does not include the RECOVERY Trial and includes one trial that we categorized as non-randomized [69]. The other meta-analysis, authored by some members of our team [64] included four peer-reviewed articles, five preprints and the RECOVERY Trial press release and showed no statistically significant benefits for mortality or other clinical outcomes. The project described here is the only meta-analysis with a collaborative approach that captures ongoing randomized trial evidence regardless of status. We regard our design as complementary to traditional systematic reviews. Whereas comprehensive inclusion of results unavailable through traditional venues may be helpful in evidence synthesis [70, 71], non-peer-reviewed results should be viewed with more caution. This trade-off between quality control and results availability may become a more pressing issue as preprints are becoming a more popular means of disseminating clinical trial results [72]. This review incorporates yet another dimension by including data from ongoing trials, some of which may be unable to achieve their planned sample size or that may go unreported.

We encourage trial investigators to coordinate early on in the design and conduct of their RCTs. Beyond providing evidence for clinical decision making, such an approach can foster evidence-based research and strategic evidence generation in situations where several trial teams address the same urgent research questions. Collaborative meta-analyses of ongoing trials do not provide final evidence but could be crucial to guide clinical decisions as well as data steering committee decisions.

Several limitations with our review should be considered. First, we only examined mortality. However, all-cause mortality in hospitalized COVID-19 patients is arguably the most important patient-relevant outcome in this setting; can be reliably measured; is most robust against sources of bias; and can be rapidly collected from diverse trials without complex data harmonization. Consistent results in the subset of placebo-controlled trials, the fact that attrition was overall negligible, and that all trials were randomized (as confirmed for all unpublished trials by investigators) further corroborated that the overall risk of bias within the trials is probably not high. However, it cannot be ruled out that the self-selected response from unpublished trial teams may introduce a reporting bias, e.g. if willingness to contribute data to the collaborative analysis depended on the results of interim trial analyses, as suggested by the Egger’s test. Nevertheless, the potential reporting bias is unlikely to change our interpretation of the results as we believe that small studies with null results were less likely to be shared with us and would contribute little to the overall evidence.

Second, we had limited ability to address potential effect modification by the timing, dose, or titer for plasma treatment. We also did not collect detailed information on various patient characteristics, including age, sex, comorbidities, and concomitant treatment (including dexamethasone) disclosed in individual trial publications which would allow further insights on potentially smaller or greater benefits in certain subgroups. For example, the trials in this meta-analysis did not specifically study patients with B-cell depletion or other immunodeficiencies. Moreover, as the participants in all included trials except one were hospitalized at enrollment, representing a group with moderate to critical COVID-19, results have unclear applicability to outpatients. According to our search, nine outpatient trials (one terminated, seven with ongoing recruitment and one not yet started) are in the pipeline. In their updated emergency use authorization, the US FDA restricted the authorization to the use of high-titer plasma in hospitalized patients early in the course of the disease [34]. While early administration of high-titer plasma has been advocated also elsewhere [1], only a small minority of RCTs have applied this regimen. Four of 32 RCTs here included used early administration of high-titer plasma in hospitalized patients (ASCOT, ConPlas-19, REMAP-CAP, and NCT04392414), and one in outpatients (NCT04479163), and our study-level analysis did not find subgroup effects regarding titer or timing. Although our definition of high-titer was chosen to conform with US FDA guidance, there is still a limited amount of comparative data between assays used in different countries (and in some cases, individual working groups) to translate titer levels. It cannot be excluded that patients treated earlier within the onset of symptoms, or with milder COVID-19, may benefit from treatment with convalescent plasma. As the majority of included RCTs are ongoing, they are expected to contribute more evidence in the coming months, together with additional evidence from individual patient data meta-analyses, e.g., the COMPILE project [49]. This may shed light on important outcomes other than all-cause mortality (e.g., severe respiratory disease or hospitalization rate), as well as possible subgroup effects such as early administration of high-titer plasma.

Third, our subgroup analyses in some cases made use of arbitrary categorizations, albeit chosen to be consistent with clinical practice, such as for plasma antibody titers and the timing of treatment initiation. The specification of subgroups was published in the protocol before data were obtained and analyzed. We consider all subgroup analyses exploratory and caution is warranted in interpreting such results.

Fourth, although representing a collaboration across many different countries, no data from any low-income countries were available, potentially limiting the applicability of our findings to these specific settings. Among all identified potentially eligible trials, one was situated in a low-income country (COVIDIT, Uganda; registered as NCT04542941).

Finally, even though this kind of collaborative meta-analysis relies on a detailed protocol with prespecified analyses aiming to ensure its integrity and validity, a few amendments were necessary. First, we realized the risk of bias assessment was readily feasible and added it post-hoc. Second, we did not specify a follow-up time point for the outcome assessment. We retained the latest one communicated to us; all updates requests were made systematically to all teams. Finally, we added some non-prespecified analyses and these are stated as such.

## Conclusions

Treatment with convalescent plasma for COVID-19 was not shown to reduce mortality and confidence intervals excluded a meaningful clinical effect. These results provide strong evidence that convalescent plasma treatment for patients with COVID-19 should not be used outside of randomized trials. Evidence synthesis from collaborations among trial investigators can inform both evidence generation and evidence application in patient care.

## Supplementary Information

**Additional file 1.** Search strategy.

**Additional file 2.** Email invitation.

**Additional file 3.** Amendments to the protocol.

**Additional file 4.** Identified potentially eligible trials not included in the analysis.

**Additional file 5.** Risk of bias.

**Additional file 6.** Funnel plot.

**Additional file 7.** Sensitivity analyses: various meta-analytic approaches.

## Data Availability

The dataset used and/or analyzed during the current study is  available at the Open Science Framework: https://osf.io/gr8jt/.

## Abbreviations

COVID-19: Coronavirus 19
EUA: Emergency use authorization
FDA: US Food and Drug Administration
HKSJ: Hartung–Knapp–Sidik–Jonkman
HLA: Human leukocyte antigen
ICU: Intensive care unit
IgG: Immunoglobulin G
LSR: Living systematic review
PM: Paule and Mandel
RBD: Receptor binding domain
RCT: Randomized clinical trial
SARS-CoV-2: Severe Acute Respiratory Syndrome Coronavirus
S/C value: Signal-to-cutoff value
