# Supplementary material for: Association between convalescent plasma treatment and mortality in COVID-19: a collaborative systematic review and meta-analysis of randomized clinical trials
Source: BMC Infect Dis. 2021 Nov 20;21:1170. doi: 10.1186/s12879-021-06829-7 (PMC8605464; doi:10.1186/s12879-021-06829-7)
Supplement: Supplementary file 1 — Additional file 1. Search strategy. [file 12879_2021_6829_MOESM1_ESM.docx]

**Additional file 1. Search strategy**

The COVID-evidence database includes trials registered on ClinicalTrials.gov or the WHO International Clinical Trials Registry Platform up to September 28, 2020, as well as trials posted or published at the following sites up to April 9, 2020: PubMed, medRxiv, bioRxiv, the WHO COVID-19 literature database, and a listing of all trials with ethical approval in Switzerland (for details please see the COVID-evidence study protocol on the Open Science Framework:<http://dx.doi.org/10.17605/OSF.IO/GEHFX>). This supplementary information describes the search strategy used to complement the COVID-evidence database with trials registered, posted or published after April 9, 2020.

**1. PubMed**

PubMed was searched from inception to April 8, 2021. Search terms for PubMed included extensive controlled vocabulary and Medical Subject Headings (MeSH):

#1 corona[ti] OR covid*[ti] OR sars[ti] OR severe acute respiratory syndrome[ti] OR ncov*[ti] OR "severe acute respiratory syndrome coronavirus 2" [Supplementary Concept] OR "COVID-19" [Supplementary Concept] OR (wuhan[tiab] AND coronavirus[tiab]) OR (wuhan[tiab] AND pneumonia virus[tiab]) OR COVID19[tiab] OR COVID-19[tiab] OR coronavirus 2019[tiab] OR SARS-CoV-2[tiab] OR SARS2[tiab] OR SARS-2[tiab] OR "severe acute respiratory syndrome 2"[tiab] OR 2019-nCoV[tiab] OR (novel coronavirus[tiab] AND 2019[tiab]) NOT (animals[mesh] NOT humans[mesh]) AND ("2019/12/01"[EDAT] : "3000/12/31"[EDAT])

#2 ((((plasma[MeSH Terms]) OR (serum[MeSH Terms])) OR (plasma[Title/Abstract])) OR (serum[Title/Abstract])) OR (convalescen*[Title/Abstract])

# 3 (randomized controlled trial[pt] OR controlled clinical trial[pt] OR randomized[tiab] OR placebo[tiab] OR clinical trials as topic[mesh:noexp] OR randomly[tiab] OR trial[ti] NOT (animals[mh] NOT humans [mh]))

#1 AND #2 AND #3

**2. Cochrane COVID-19 trial registry**

The Cochrane COVID-19 trial registry (<https://covid-19.cochrane.org/>) was searched from inception to April 8, 2021. Search terms were “convalescent OR plasma”, and we used the filter categories “Intervention assignment” (“randomised”), “Study aim” (“Treatment and management”), and “Study type” (“Intervention”).

**3. L·OVE Platform - Epistemonikos**

The L·OVE Platform (<https://iloveevidence.com/>) was searched from inception to April 8, 2021. We used the filter categories “Prevention and treatment”, “Procedures - convalescent plasma”, and within the primary studies we selected the filters “by reported data - has data” and “by type of study - RCT”.

**4. Other sources**

Finally, we complemented our results with trials identified by other published or registered systematic searches as well as personal knowledge.
