# Supplementary material for: Association between convalescent plasma treatment and mortality in COVID-19: a collaborative systematic review and meta-analysis of randomized clinical trials
Source: BMC Infect Dis. 2021 Nov 20;21:1170. doi: 10.1186/s12879-021-06829-7 (PMC8605464; doi:10.1186/s12879-021-06829-7)
Supplement: Supplementary file 2 — Additional file 2. Email invitation. [file 12879_2021_6829_MOESM2_ESM.docx]

**Additional file 2. Email invitation**

Subject: Co-author invitation, international collaborative meta-analysis on convalescent plasma and mortality in COVID-19

Send to:

«Contact_email»; «Contact_email_2»

Dear «Title» «Contact_last_name» «and_» «Contact_last_name_2»,

We would like to invite your trial investigation team to **collaborate as co-authors on a rapid international meta-analysis** on the effect of convalescent plasma on mortality in COVID-19 in randomized controlled trials (RCTs), together with our collaborative research group coordinated at Stanford University, USA, and the University of Basel, Switzerland. Your study «Acronym_or_title» is highly relevant for this project.

As you are aware, the US Food and Drug Administration (FDA) issued an emergency use authorization for convalescent plasma treatment in COVID-19 on August 23, although randomized evidence is very limited. Our searching suggests that almost 90 RCTs are ongoing; however, no single trial is projected to yield definitive results regarding mortality in 2020. Therefore, we are sending this invitation to investigators of all ongoing, discontinued or completed RCTs that evaluate convalescent plasma treatment for COVID-19 against placebo or no treatment. **With your help, we are hoping to provide a rapid summary of the current evidence using a meta-analysis of group-level, aggregated, all-cause mortality data (NOT individual patient data).** We aim to rapidly publish the results in a peer-reviewed journal and your core team is invited to co-author the publication.

We have no commercial interest with this work, and our protocol can be found attached as well as registered on the Open Science Framework: [link]. Trials that are eligible for this project are listed in the protocol (Supplement 3, last page).

**As collaborators, we kindly ask you to answer the following questions before October 25**. If you are interested in collaborating but uncertain whether the data may be shared before October 25, please respond as soon as possible.

Question 1: Could you please confirm that these criteria apply to your trial?

a) The trial is randomized

b) The trial has at least one group of patients who receive convalescent plasma

c) The trial has at least one control group that does NOT receive convalescent plasma

Question 2: For each of your study arms,

a) What intervention did this group receive?

b) How many patients were randomized to this group?

c) Of these patients, how many have died?

d) Of these patients, for how many it is unknown if they are dead or alive?

Once we have completed the required data analyses, a draft manuscript will be shared with all co-authors for comments. We will also ask you to confirm or complement the trial design characteristics listed in the protocol for potential subgroup analyses (attached). The finalized version will be submitted to a peer-reviewed medical journal.

From our perspective, this initiative does of course NOT stand in conflict with taking part in other collaborations or publishing your trial results individually. The focus of this meta-analysis is limited to all-cause mortality. It does not include other outcomes, and it will not use individual patient data. With this publication we aim to make sure that all clinical trial data hitherto collected (unpublished or published) will be of use, regardless of whether the target sample size of each trial was reached or not. If you know of other collaborations, we would be thankful for contact information so that we may coordinate and optimize our efforts.

Thank you for considering our request and please do not hesitate to ask for clarifications.

Kind regards,

Lars Hemkens, Steve Goodman, David Moher, John Ioannidis, Cathrine Axfors, Perrine Janiaud, and Andreas Schmitt for the COVID-evidence team

Our COVID-evidence database (www.covid-evidence.org) is used for this work, supported by the Swiss National Science Foundation (Project ID 196190) and a large collaboration of researchers from Switzerland, USA, China, Canada, UK, France, Germany, Austria, Sweden, the Netherlands, and other countries.
