# Supplementary material for: Association between convalescent plasma treatment and mortality in COVID-19: a collaborative systematic review and meta-analysis of randomized clinical trials
Source: BMC Infect Dis. 2021 Nov 20;21:1170. doi: 10.1186/s12879-021-06829-7 (PMC8605464; doi:10.1186/s12879-021-06829-7)
Supplement: Supplementary file 3 — Additional file 3. Amendments to the protocol. [file 12879_2021_6829_MOESM3_ESM.docx]

**Additional file 3. Amendments to the protocol**

| Protocol v.1.1^a^ description/section | Amendment | Rationale |
| --- | --- | --- |
| We specified the search date to September 28, 2020. We did not specify a date for inviting investigators of eligible trials of which the results were not publicly available. | The initial search was performed September 28, 2020, and investigators of eligible but unavailable trials were contacted shortly after.  An updated search was done by March 1, 2021, and investigators of eligible but unavailable trials were contacted also then.  Final search update was done on April 8, 2021. | Given the dynamic nature of this kind of collaboration, and to ensure maximal participation by trial investigators, we did not set a definitive end date for data collection. |
| Did not state how to choose between several potential follow-up points in the same study | If several follow-up points were available, we chose the longest. | We considered the longest available follow-up to be most informative for the study question. |
| Did not include risk of bias assessment of individual studies | Two reviewers (CA and PJ) independently assessed the risk of bias of included RCTs using the Cochrane risk of bias tool 2.0. Disagreements were resolved through discussion. The assessment was done using information reported in the preprints and journal publications or provided by investigators for unpublished trials. | In line with recommended practice. |
| Subgroup analyses | (1) We do not report the prespecified subgroup analysis “Loss to follow-up (more than 10% of randomized patients versus 10% or less)  (2) We added a subgroup analysis stratifying trials into high-income countries and middle-income countries.  (3) We also added a non-prespecified subgroup analysis separating trials with early administration of high-titer plasma in hospitalized patients from other trials.  (4) We applied Hartung-Knapp “ad hoc” variance correction for the subgroup analysis on donor pregnancy status ( “Excluding potentially HLA antibody positive persons”).  (5) We complemented the high-titer definition with additional information made available in the March 2021 version US FDA emergency use authorization (e.g., EUROIMMUN (ratio ≥ 3.5) and Abbott ARCHITECT (S/C ≥ 4.5). | (1) No trials were in the former category and loss to follow-up was extremely low overall.  (2) In reflection of the geographic diversity of the trials.  (3) Given the updated emergency use authorization by the United States Food and Drug Administration in February 2021.  (4) This group included only two very small studies with large imprecision which can provide abnormally anticonservative estimates  (5) In accordance with the latest information provided by the US FDA. |
| Additional analyses | Two cumulative meta-analyses were added. | To explore and describe the accumulation of evidence. |

^a^ Janiaud P, Axfors C, Saccilotto R, Hemkens L. COVID-evidence: a living database of trials on interventions for COVID-19. 2020 Apr 1; Available from: https://osf.io/gehfx/
