# Supplementary material for: Association between convalescent plasma treatment and mortality in COVID-19: a collaborative systematic review and meta-analysis of randomized clinical trials
Source: BMC Infect Dis. 2021 Nov 20;21:1170. doi: 10.1186/s12879-021-06829-7 (PMC8605464; doi:10.1186/s12879-021-06829-7)
Supplement: Supplementary file 4 — Additional file 4. Identified potentially eligible trials not included in the analysis. [file 12879_2021_6829_MOESM4_ESM.docx]

**Additional file 4. Identified potentially eligible trials not included**

| Acronym, registration number or title | Experimental treatment | Control treatment | Setting | Location | Arms | Target sample size | Contact name |
| --- | --- | --- | --- | --- | --- | --- | --- |
| CSSC-004 | Convalescent plasma | Placebo | Outpatient | USA | 2 | 1344 | Sullivan |
| CONCOR-1 | Convalescent plasma | Standard of care | Inpatient | Canada and USA | 2 | 1200 | Arnold |
| RES-Q-HR (registered as NCT04681430) | Convalescent plasma | Standard of care | Outpatient | Germany | 4 | 1094 | Keitel-Anselmino |
| NCT04649879 | Convalescent plasma + standard of care | Standard of care | Inpatient | Sweden | 2 | 920 | Dillner |
| VA CURES-1 | Convalescent plasma | Placebo | Inpatient | USA | 2 | 702 | Janoff |
| CoV-Early Study (registered as NCT04589949) | Convalescent plasma | Placebo | Outpatient | Netherlands | 2 | 690 | Rijnders |
| PROTECT-Patient study | Convalescent plasma + standard of care | Placebo + standard of care | Inpatient | South Africa | 2 | 600 | Nyoni |
| C3PO | Convalescent plasma | Placebo | Outpatient | United States | 2 | 600 | Schulman |
| PassItOnII | Convalescent plasma | Placebo | Inpatient | United States | 2 | 500 | Bistran-Hall |
| DAWN-Plasma | Convalescent plasma + standard of care | Standard of care | Inpatient | Belgium | 2 | 483 | Meyfroidt |
| Registered as NCT04621123 | Convalescent plasma + standard of care | Placebo + standard of care | Outpatient | Spain | 2 | 474 | Mitjà |
| PLATINA TRIAL | Convalescent plasma | Standard of care | Inpatient | India | 2 | 472 | Meshram |
| COV-PLAS | Convalescent plasma | Placebo | Inpatient | Netherlands | 2 | 430 | Zwaginga |
| EPCOvid-1 | Convalescent plasma | Placebo | Inpatient | Mexico | 2 | 410 | Sierra-Madero |
| ILBS-COVID-04 | Convalescent plasma + standard of care | Standard of care | Inpatient | India | 2 | 400 | Bajpai |
| COV2-CP | Convalescent plasma + standard of care | Standard of care | Inpatient | Italy | 2 | 400 | Talarico |
| Registered as NCT04456413 | Convalescent plasma | Standard of care | Outpatient | United States | 2 | 306 | Vendivil |
| CONTAIN COVID-19 | Convalescent plasma | Placebo | Inpatient | United States | 2 | 300 | Ortigoza |
| PLASMA COVID-19  registered as NCT04425837 | Convalescent plasma + standard of care | Standard of care | Inpatient | Colombia | 2 | 236 | Quintero-Vega |
| ESCAPE | Convalescent plasma | Placebo | Inpatient | United States | 2 | 220 | Kaufman |
| PLAVID-TRIAL | Convalescent plasma + standard of care | Standard of care | Inpatient | Brazil | 2 | 220 | NA |
| Registered as PACTR202007653923168 | Convalescent plasma + standard of care | Standard of care | Inpatient | Kenya | 2 | 206 | Adembesa |
| Registered as NCT04390503 | Convalescent plasma | Placebo | Outpatient | United States | 4 | 200 | Justman |
| IMPACT | Convalescent plasma + standard of care | Placebo + standard of care | Inpatient | Ecuador | 2 | 200 | Baldeon |
| PLACOVID | NA | NA | NA | Brazil | 2 | 160 | Sekine |
| MIDC-CCP | Convalescent plasma | Standard of care | Outpatient | United States | 2 | 150 | Van Hise |
| PC-COVID-HCM | NA | NA | NA | Mexico | 2 | 150 | Torres |
| COVIDIT | NA | NA | NA | Uganda | 2 | 136 | NA |
| TSUNAMI^a^ | Convalescent plasma + standard of care | Standard of care | Inpatient | Italy | 2 | 126 | Falcone |
| COOPCOVID-19 | Convalescent plasma + standard of care | Standard of care | Inpatient | Brazil | 3 | 120 | Nakagawa |
| CORIPLASM | Convalescent plasma | Standard of care | Unclear | France | 2 | 120 | Lacombe |
| Registered as IRCT20200501047258N1 | Convalescent plasma | Standard of care | Inpatient | Iran | 3 | 120 | Asghari |
| Registered as NCT04333251 | Convalescent plasma | Standard of care | Inpatient | United States | 2 | 115 | Baylor Research Institute team |
| Registered as ChiCTR2000030179 | Convalescent plasma + standard of care | Standard of care | Inpatient | China | 2 | 100 | Le Aiping |
| Registered as ChiCTR2000030010 | Convalescent plasma | Ordinary plasma | Inpatient | China | 2 | 100 | Zhang Dingyu |
| CONCOR-KIDS | Convalescent plasma + standard of care | Standard of care | Inpatient | Canada | 2 | 100 | Upton |
| MHC-COVID-19-CP | Convalescent plasma | Standard of care | Inpatient | India | 2 | 100 | Pathak |
| Registered as IRCT20120215009014N353 | Convalescent plasma + standard of care | Standard of care | Inpatient | Iran | 2 | 100 | Ghelichkhani |
| COVID PLASMA STUDY | Convalescent plasma | Standard of care | Inpatient | India | 2 | 100 | Chawla |
| Registered as NCT04547127 | NA | NA | NA | Spain | 2 | 100 | Torres |
| PennCCP-02 | Convalescent plasma + standard of care | Standard of care | Inpatient | United States | 2 | 80 | Bar |
| PLASCOSSA | NA | Placebo | Inpatient | France | 2 | 80 | Martinaud |
| LFCOLCOVID-19-001 | Convalescent plasma + standard of care | Standard of care | Inpatient | Colombia | 3 | 75 | Jaramillo |
| IRCT20201004048922N1 | Convalescent plasma + standard of care | Standard of care | Inpatient | Iran | 3 | 75 | Abtahi |
| Registered as ChiCTR2000030929 | Convalescent plasma | Ordinary plasma | Inpatient | China | 2 | 60 | Binghong Zhang |
| Registered as NCT04442958 | Convalescent plasma + standard of care | Standard of care | ICU | Turkey | 2 | 60 | NA |
| Registered as NCT04380935 | Convalescent plasma + standard of care | Standard of care | Inpatient | Indonesia | 2 | 60 | Sinto |
| Registered as NCT04385186 | Convalescent plasma + standard of care | Standard of care | Inpatient | Colombia | 2 | 60 | Zuluaga |
| Registered as IRCT20200404046948N1 | Convalescent plasma + standard of care | Standard of care | Inpatient | Iran | 2 | 60 | Farahani |
| Registered as CTRI/2020/05/025346 | Convalescent plasma + Standard of care | Standard of care | Inpatient | India | 2 | 60 | Kuzhali |
| Registered as ChiCTR2000030702 | Convalescent plasma + standard of care | Standard of care | Inpatient | China | 2 | 50 | Cao Bin |
| Registered as NCT04421404 | Convalescent plasma | Placebo | Inpatient | United States | 2 | 50 | Park |
| CPCP | Convalescent plasma + standard of care | Standard of care | Inpatient | Vietnam | 2 | 44 | Phuong Hoang Nguyen |
| Registered as IRCT20200503047281N1 | Convalescent plasma + standard of care | Standard of care | Inpatient | Iran | 2 | 40 | Hadinedoushan |
| Not registered | Convalescent plasma | Standard of care | Outpatient and inpatient | Germany | NA | 40 | Meybohm |
| Registered as ChiCTR2000030627 | Convalescent plasma + standard of care | Standard of care | Inpatient | China | 2 | 30 | Guojun Zhang |
| Registered as NCT04530370 | Convalescent plasma | Placebo | Inpatient | Egypt | 2 | 30 | Salman |
| PROMETEO | Convalescent plasma + standard of care | Placebo + standard of care | ICU | Mexico | 2 | 15 | Martinez |
| Registered as IRCT20200413047056N1 | Convalescent plasma + standard of care | Standard of care | Inpatient | Iran | 3 | 15 | Zangoue |
| PER-060-20 | NA | NA | NA | NA | NA | NA | NA |
| Registered as NCT04391101^a^ | Convalescent plasma + standard of care | Standard of care | ICU | Colombia | 2 | 231 | Perilla |
| "Randomized pilot study for the use of convalescent plasma in patients with COVID-19 disease"^a^ | Convalescent plasma | Standard of care | ICU | Spain | NA | 60 | Cid |
| Not registered^a^ | NA | NA | NA | Norway | NA | NA | Hervig |
| Registered as NCT04467151^a^ | Convalescent plasma | Placebo | Inpatient | United States | 2 | 96 | McIntee |
|  |  |  |  |  |  |  |  |
| CONFIRMED INELIGIBLE | | | | | | | |
| Registered as NCT04375098^b^ | Convalescent plasma | Standard of care (convalescent plasma if deterioration) | Inpatient | Chile | 2 | 30 | Balcells |
| Registered as IRCT20150808023559N21^c^ | Convalescent plasma + standard of care | Standard of care | Inpatient | Iran | 2 | 60 | Jam |
| CPT-COVID-19  Registered as  NCT04441424^c^ | Convalescent plasma + standard of care | Standard of care | Inpatient | Iraq | 2 | 58 | NA |
| NCT04468009^c^ | Convalescent plasma + standard of care | Standard of care | Inpatient | Argentina | 2 | 36 | Gonzalez |
| CTRI/2020/05/025299^c^ | Convalescent plasma + standard of care | Standard of care | Inpatient | India | 2 | 20 | Bhatia |

^a^ Withdrawn. ^b^ No eligible control group. ^c^ Did not use an eligible randomization procedure.
