# Supplementary material for: Association between convalescent plasma treatment and mortality in COVID-19: a collaborative systematic review and meta-analysis of randomized clinical trials
Source: BMC Infect Dis. 2021 Nov 20;21:1170. doi: 10.1186/s12879-021-06829-7 (PMC8605464; doi:10.1186/s12879-021-06829-7)
Supplement: Supplementary file 5 — Additional file 5. Risk of bias. [file 12879_2021_6829_MOESM5_ESM.docx]

**Additional file 5. Risk of Bias**

| Study | 1. Randomization process | 2. Deviations from  the intended  interventions | 3. Missing  outcome data | 4. Measurement  of the outcome | 5. Selection of  the reported  result | Overall risk of bias |
| --- | --- | --- | --- | --- | --- | --- |
| PUBLISHED |  |  |  |  |  |  |
| ChiCTR2000029757 | Low | Low | Low | Low | Low | Low |
| ConPlas-19 | Some concerns | Low | Low | Low | Low | Some concerns^a^ |
| ILBS-COVID-02 | Low | Low | Low | Low | Low | Low |
| IRCT20200310046736N1 | Low | Low | Low | Low | Low | Low |
| NCT04342182 | Low | Low | Low | Low | Low | Low |
| NCT04356534 | Some concerns | Low | Low | Low | Low | Some concerns^a^ |
| NCT04359810 | Low | Low | Low | Low | Low | Low |
| NCT04392414 | Low | Low | Low | Low | Low | Low |
| NCT04479163 | Low | Low | Low | Low | Low | Low |
| PICP19 | Some concerns | Some concerns | Low | Low | Low | High risk^b^ |
| PLACID | Low | Low | Low | Low | Low | Low |
| PLASM-AR | Low | Low | Low | Low | Low | Low |
| RECOVERY | Low | Low | Low | Low | Low | Low |
| UNPUBLISHED |  |  |  |  |  |  |
| ASCOT | Low | Low | Low | Low | Low | Low |
| CAPSID | Low | Low | Low | Low | Low | Low |
| CCAP-2 | Low | Low | Low | Low | Low | Low |
| Co-CLARITY | Low | Low | Low | Low | Low | Low |
| CONFIDENT | Low | Low | Low | Low | Low | Low |
| COP-COVID-19 | Some concerns | Low | Low | Low | Low | Some concerns^c^ |
| CPC-SARS | Low | Low | Low | Low | Low | Low |
| LACCPT | Low | Low | Low | Low | Low | Low |
| LIFESAVER | Low | Low | Low | Low | Low | Low |
| NCT04332835 | Low | Low | Low | Low | Low | Low |
| NCT04385199 | Low | Low | Low | Low | Low | Low |
| NCT04403477 | Low | Low | Low | Low | Low | Low |
| NCT04442191 | Low | Low | Low | Low | Low | Low |
| NCT04528368 | Low | Low | Low | Low | Low | Low |
| NCT04600440 | Low | Low | Low | Low | Low | Low |
| PC/COVID-19 | Low | Low | Low | Low | Low | Low |
| PERUCONPLASMA | Low | Low | Low | Low | Low | Low |
| PLACO-COVID | Low | Low | Low | Low | Low | Low |
| RECOVER | Low | Low | Low | Low | Low | Low |
| REMAP-CAP | Low | Low | Low | Low | Low | Low |

*^a^ Concerns as no detailed information regarding (i) the randomization and (ii) the randomization concealment reported*

*^b^ Concerns as no detailed information regarding (i) the randomization and (ii) the randomization concealment reported. Further concerns regarding deviations from the intended interventions due to the open label setting of the study with missing information regarding the flowchart of patients and potential deviations*

*^c^ Concerns as unclear if the randomization was done centrally ensuring the randomization concealment*

*Of note, NCT04392414 included retrospectively 2 patients without randomizing them in the control arm. As it only represents 3% of the randomized patients (n=66) it is unlikely to affect the assessment of the effect of the intervention.*
