# Supplementary material for: Association between convalescent plasma treatment and mortality in COVID-19: a collaborative systematic review and meta-analysis of randomized clinical trials
Source: BMC Infect Dis. 2021 Nov 20;21:1170. doi: 10.1186/s12879-021-06829-7 (PMC8605464; doi:10.1186/s12879-021-06829-7)
Supplement: Supplementary file 6 — Additional file 6. Funnel plot. [file 12879_2021_6829_MOESM6_ESM.docx]

**Additional file 6. Funnel plot**

Egger’s test p value = 0.0455

The dashed vertical line denotes the log of the overall risk ratio of 0.97.
