# Supplementary material for: Association between convalescent plasma treatment and mortality in COVID-19: a collaborative systematic review and meta-analysis of randomized clinical trials
Source: BMC Infect Dis. 2021 Nov 20;21:1170. doi: 10.1186/s12879-021-06829-7 (PMC8605464; doi:10.1186/s12879-021-06829-7)
Supplement: Supplementary file 7 — Additional file 7. Sensitivity analyses: various meta-analytic approaches. [file 12879_2021_6829_MOESM7_ESM.docx]

**Additional file 7. Sensitivity analyses: various meta-analytic approaches**

|  | **Multi-arms trial kept separately** | | **Multi-arms trials pooled** | |
| --- | --- | --- | --- | --- |
|  | **Reciprocal of contrasting arm added to zero events**  **RR (95% CI), I², Tau²** | **Arcsine difference**  **ASD (95% CI), I², Tau²** | **Reciprocal of contrasting arm added to zero events**  **RR (95% CI), I², Tau²** | **Arcsine difference**  **ASD (95% CI), I², Tau²** |
| **HKSJ-PM** | 0.97 (0.92, 1.02); 0, 0 | -0.02 (-0.05, 0.01); 0.07, 0 | 0.97 (0.92, 1.02); 0, 0 | -0.03 (-0.06, 0.01); 0.1, 0 |
| **HKSJ-SJ** | 0.84 (0.7, 1.01); 0, 0.24 | -0.04 (-0.08, 0); 0.07, 0.01 | 0.84 (0.7, 1.02); 0, 0.25 | -0.04 (-0.09, 0); 0.1, 0.01 |
| **MH-DSL** | 0.97 (0.92, 1.02); 0, 0 | -0.02 (-0.05, 0); 0.07, 0 | 0.97 (0.92, 1.02); 0, 0 | -0.02 (-0.05, 0); 0.1, 0 |
| **PETO-OR** | 0.95 (0.87, 1.03); 0.01, 0 | -0.02 (-0.05, 0); 0.07, 0 | 0.93 (0.84, 1.04); 0.04, 0 | -0.02 (-0.05, 0); 0.1, 0 |
| **Profile likelihood** | 0.97 (0.86, 1.02); NA, 0 | -0.01 (-0.05, 0.01); NA, 0 | 0.97 (0.86, 1.02); NA, 0 | -0.01 (-0.05, 0.01); NA, 0 |

HKSJ-PM = Hartung-Knapp-Sidik-Jonkman adjustment for random effects model, Paule-Mandel estimator for tau²;

HKSJ-SJ = Hartung-Knapp-Sidik-Jonkman adjustment for random effects model, Sidik-Jonkman estimator for tau²;

MH-DSL = Mantel-Haenszel method for random effects model, DerSimonian-Laird estimator for tau²;

PETO-OR = Peto’s odd ratio method with random effects model, Paule-Mandel estimator for tau²;

Profile likelihood = Profile likelihood method with random effects model, likelihood-based confidence intervals
